# Morphometric discrimination of two sympatric sibling species in the Palaearctic region, *Culicoides obsoletus* Meigen and *C. scoticus* Downes & Kettle (Diptera: Ceratopogonidae), vectors of bluetongue and Schmallenberg viruses

**DOI:** 10.1186/s13071-016-1520-7

**Published:** 2016-05-04

**Authors:** G. Kluiters, N. Pagès, S. Carpenter, L. Gardès, H. Guis, M. Baylis, C. Garros

**Affiliations:** Liverpool University Climate and Infectious Diseases of Animals (LUCINDA) Group, Institute of Infection and Global Health, University of Liverpool, Leahurst Campus, Neston, Cheshire CH64 7TE UK; Centre de Recerca en Sanitat 26 Animal (CReSA), UAB-IRTA, Campus de la Universitat Autònoma de Barcelona, 08193 Bellaterra, Barcelona Spain; Vector-borne Viral Disease Programme, The Pirbright Institute, Woking, GU24 0NF UK; Cirad, UMR15 CMAEE, F-34398 Montpellier, France; INRA, UMR1309 CMAEE, F-34398 Montpellier, France; National Institute for Health Research, Health Protection Research Unit in Emerging and Zoonotic Infections, University of Liverpool, Liverpool, UK; Present address: CIRAD, UMR CMAEE, F-97170 Petit-Bourg, Guadeloupe France

**Keywords:** Arbovirus, Bluetongue virus, BTV, *Culicoides obsoletus*, *Culicoides scoticus*, Obsoletus Group, Obsoletus complex, Morphology, Morphometrics, Sibling species

## Abstract

**Background:**

Some Palaearctic biting midge species (subgenus *Avaritia*) have been implicated as vectors of bluetongue virus in northern Europe. Separation of two species (*C. obsoletus* and *C. scoticus*) is considered difficult morphologically and, often, these female specimens are grouped in entomological studies. However, species-specific identification is desirable to understand their life history characteristics, assess their roles in disease transmission or measure their abundance during arboviral outbreaks. This study aims to investigate whether morphometric identification techniques can be applied to female *C. obsoletus* and *C. scoticus* individuals trapped at different geographical regions and time periods during the vector season.

**Methods:**

*C. obsoletus* and *C. scoticus* were collected using light-suction traps from the UK, France and Spain, with two geographical locations sampled per country. A total of 759 *C. obsoletus*/*C. scoticus* individuals were identified using a molecular assay based on the cytochrome *c* oxidase subunit I gene. Fifteen morphometric measurements were taken from the head, wings and abdomen of slide-mounted specimens, and ratios calculated between these measurements. Multivariate analyses explored whether a combination of morphometric variables could lead to accurate species identification. Finally, *Culicoides* spp. collected in France at the start, middle and end of the adult vector season were compared, to determine whether seasonal variation exists in any of the morphometric measurements.

**Results:**

The principal component analyses revealed that abdominal characteristics: length and width of the smaller and larger spermathecae, and the length of the chitinous plates and width between them, are the most reliable morphometric characteristics to differentiate between the species. Seasonal variation in the size of each species was observed for head and wing measurements, but not abdominal measurements. Geographical variation in the size of *Culicoides* spp. was also observed and is likely to be related to temperature at the trapping sites, with smaller individuals trapped at more southern latitudes.

**Conclusions:**

Our results suggest that female *C. obsoletus* and *C. scoticus* individuals can be separated under a stereomicroscope using abdominal measurements. Although we show the length and width of the spermathecae can be used to differentiate between the species, this can be time-consuming, so we recommend undertaking this using standardized subsampling of catches.

**Electronic supplementary material:**

The online version of this article (doi:10.1186/s13071-016-1520-7) contains supplementary material, which is available to authorized users.

## Background

Biting midges of the genus *Culicoides* (Diptera: Ceratopogonidae) transmit several important viruses of ruminants, such as bluetongue virus (BTV), Epizootic Haemorrhagic Disease virus and Schmallenberg virus (SBV) as well as African horse sickness virus to equids [1]. The disease caused by BTV, called bluetongue, was considered exotic to Europe prior to 1998 [2]; subsequently, numerous serotypes have been detected in the Mediterranean basin and one in particular, serotype 8 (BTV-8), caused a significant outbreak in northern Europe that began in 2006 [3]. These outbreaks caused dramatic sanitary and economic consequences in affected countries [4]. In 2011, a novel *Culicoides*-borne disease, called Schmallenberg, was detected on European farms, causing congenital malformations and stillbirths in both cattle and sheep, as well as mild disease in adult cattle. The causative agent was found to be a novel Simbu serogroup virus from the genus *Orthobunyavirus*, SBV [5]. Although some orthobunyaviruses had previously been reported in Europe, viruses from the Simbu serogroup had not previously been isolated in the region. The recent emergence of these different diseases highlights the need for a greater understanding of the epidemiology and transmission of *Culicoides*-borne viruses in Europe. This understanding requires detailed knowledge of the vector species themselves, including their vector competence, life history characteristics and relative abundances [6, 7]. This, in turn, requires the means to accurately identify them to species level, something that is not, however, straightforward.

Since 2006, certain Palaearctic *Culicoides* biting midge species have been implicated as vectors of BTV and SBV in northern Europe [8, 9]; of particular importance are members of the subgenus *Avaritia*, namely *Culicoides obsoletus* Meigen*, C. scoticus* Downes & Kettle, *C. dewulfi* Goetghebuer and *C. chiopterus* Meigen. The four species are difficult to separate under a light microscope and, therefore, alternative methods have been developed [6, 10]. Multiplex PCR assays are most commonly used, based upon the cytochrome *c* oxidase subunit I (COI), internal transcribed spacer 1 (ITS-1) and the internal transcribed spacer 2 genes (ITS-2) [10–16]. A recent ring trial study has demonstrated the higher specificity of the COI-based assay from Nolan et al. [10] compared to other one-step species-specific molecular identification assays [17].

In many situations it is not practical to rely on molecular methods of identification of *Avaritia* group *Culicoides*, particularly for large scale studies, as the methods are time-consuming and can be expensive. As a result, females (either *C. obsoletus* and *C. scoticus*, or all four species) are often grouped as a single entity in both small and large entomological surveys [18–23]. Separation of the species under the microscope remains desirable, therefore, in many situations, especially as this can be undertaken at the same time as *Culicoides* are separated from other insects in the collection.

Morphological identification relies on the form and structure of *Culicoides* to identify a species, often using binary keys. Important morphological characteristics include pigmentation patterning of the wings, shape of antennal segments, characteristics of the genitalia in males, distribution of the sensillae on the antennae, and the number and size of the spermathecae in females [24–29]. It is straightforward to discriminate morphologically between the males (not involved in disease transmission) of the four sympatric *Avaritia* species mentioned previously, on the basis of the shape of their hypopygium [24]. It is not straightforward, however, to discriminate morphologically between the females. Some researchers claim that the females of *C. dewulfi* and *C. chiopterus* can be differentiated: *C. chiopterus* is a smaller species than the others and the wings of both it, and *C. dewulfi*, are paler in their markings than for *C. obsoletus* and *C. scoticus*, with *C. chiopterus* in particular almost devoid of colour [30]. A pale spot at the distal end of the wing, as well as the pronounced difference in size between the spermatheca seen in *C. dewulfi* specimens, further differentiate the two species. *Culicoides obsoletus* and *C. scoticus,* however, show no distinguishing markings on their wings to aid in their differentiation. Their spermathecae are also of similar sizes [31]. Separation of *C. obsoletus* and *C. scoticus* is considered particularly difficult, if not impossible, when undertaken morphologically [32].

Morphometric discrimination, the quantitative analysis of form (e.g. measurement of the length of wings, antennae or spermathecae), is often used to separate morphologically similar species. According to Delécolle, *C. obsoletus* and *C. scoticus* females can be distinguished based on the length of the larger of their two functional spermathecae [26], although this finding was not confirmed in a later study undertaken by Pagès & Sarto I Monteys [33]. Augot et al. explored the use of 15 morphometric variables to distinguish the two species and found that females of *C. obsoletus* and *C. scoticus* can be accurately distinguished based on the width between their chitinous plates, the length and width of their larger spermatheca and the length of their smaller spermatheca [31]. More recently it has been claimed that it is possible to separate females of *C. dewulfi, C. chiopterus*, *C. obsoletus* and *C. scoticus*, by combining the shape of the third segment of the maxillary palp and the number and location of hairs on the first abdominal tergite [30]. A new approach, based on geometric morphometric analysis, has been recently implemented in the genus *Culicoides* to investigate wing shape for species identification [34–36].

A major limitation in the use of morphological or morphometric identification methods for these four species is that they have mainly been developed on populations from within one region or country and therefore do not take into account any variation in individual species between countries. The *Culicoides* spp. used in these studies have also only been collected from one time point during the year, or grouped from seasonal surveillance schemes carried out across the year, obscuring any important seasonal variation. This ignores the potential for morphological or morphometric variation according to the time of emergence.

This paper investigates whether there are morphological and morphometric identification techniques for the separation of female *C. obsoletus* and *C. scoticus* individuals that are applicable for specimens trapped in different geographical regions and at different time periods during the adult flight season.

Specific objectives included re-evaluating morphometric measurements and ratios that were previously explored by Delécolle, Pagès & Sarto I Monteys, Augot et al. and Nielsen & Christensen [26, 30, 31, 33] using *Culicoides* spp. from different countries and trapped at known time points within the adult flight season. The aim was to determine whether a combination of variables could be used to discriminate between the two species using multivariate analyses.

## Methods

### Field sampling

**Fig. 1:**
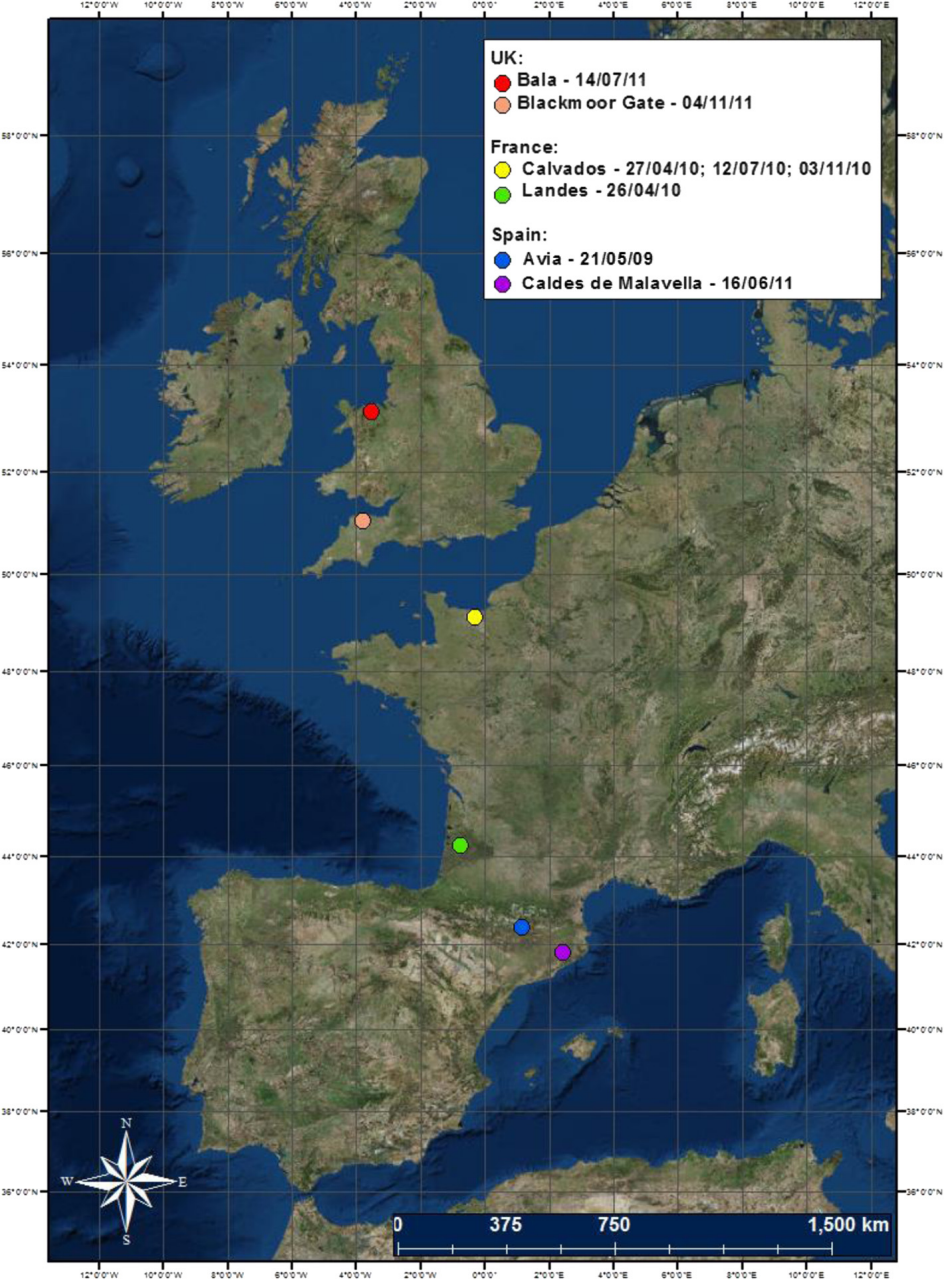
Location of field-trapping sites for *Culicoides* samples used for morphometric and molecular identification, in France (Calvados sampled 27^th^ April, 12^th^ July and 3^rd^ November 2010; Landes 26^th^ April 2010), Spain (Caldes de Malavella sampled 16^th^ June 2011; Avià 21^st^ May 2009) and the UK (Bala sampled 14^th^ July 2011; Devon 4^th^ November 2011)

Insects were sampled from two different sites in each of the UK, France and Spain between May 2009 and November 2011 (Fig. 1). In the UK, one site was a farm in the Bala region of north Wales, with *Culicoides* collected on the 14^th^ July 2011 using an Onderstepoort down-draught black light (OVI) trap [37]. The second site was a farm in Blackmoor Gate, Devon (4^th^ November 2011), where the *Culicoides* were collected using a UV-LED CDC (Centers for Disease Control) trap (John W. Hock Company, Gainesville, FL, USA). In France, the *Culicoides* from both sites were trapped using an OVI trap. One site was located on a farm in Calvados, north-western France, and *Culicoides* were taken from three trapping periods to sample the start (27^th^ April 2010), middle (12^th^ July 2010) and end (3^rd^ November 2010) of a trapping season. The *Culicoides* from the second site in Landes, south-western France, were trapped on the 26^th^ April 2010. In Spain, UV-CDC traps were used to sample *Culicoides* from Caldes de Malavella (north-eastern Spain) on 16^th^ June 2011, and Avià (northern Spain) on 21^st^ May 2009. *Culicoides* were stored in 70 % ethanol prior to morphological and molecular analyses.

### Specimen identification and mounting

*Culicoides* spp. were separated from other insects according to their wing characteristics using a stereomicroscope, before being identified as *C. obsoletus* and *C. scoticus,* or *C. dewulfi* and *C. chiopterus*. The females of *C. obsoletus*/*C. scoticus* were differentiated from *C. dewulfi*/*C. chiopterus* on the basis of spermathecae size and wing pattern, before a total of 994 *C. obsoletus* and *C. scoticus* specimens were randomly selected from the trapping sites and dates. The individual females were dissected on a slide using sterile dissecting needles (Watkins & Doncaster, Leominster, UK). The head (dorsal side up), wings and posterior abdominal segment (ventral side up) of each of these specimens were subsequently mounted on the slide under three separate cover slips using Canada balsam. The remaining thorax, legs and anterior abdomen were stored in 75 % ethanol for DNA analysis.

### Molecular identification

Prior to DNA extraction, the dissected *Culicoides* were individually removed from their ethanol-filled storage vials and placed on absorbent paper, to remove excess ethanol. *Culicoides* were added to a Macherey Nagel round well block with 500 μl of 5 % Chelex® 100 resin (Bio-Rad Laboratories, Inc., Hercules, CA, USA). Lysis was performed using 3 mm Qiagen tungsten carbide beads in two cycles of 30 agitations per second for 30 s. The beads were removed, and extraction of DNA was achieved by incubating the *Culicoides* at 56 °C for 1 h (700 rpm), then 30 min at 96 °C (650 rpm) in the 500 μl Chelex resin suspension, using an Eppendorf Thermomixer Compact.

Primers and PCR amplification conditions were as described by Nolan et al*.* [10], with four forward primers, in order to identify human error during morphological identifications:

- *C. obsoletus:* UOAobsF (5′-TGCAGGAGCTTCTGTAGATTTG-3′);
- *C. scoticus:* UOAscoF (5′-ACCGGCATAACTTTTGATCG-3′);
- *C. chiopterus:* UOAchiF (5′-TACCGCCCTCTATCACCCTA-3′);
- *C. dewulfi:* UOAdewF (5′-ATACTAGGAGCGCCCGACAT-3′); and one reverse primer:
- C1-N-2191 (5′-CAGGTAAAATTAAAATATAAACTTCTGG-3′) (Dallas et al., 2003).

The PCR of the mitochondrial COI gene was performed in a total volume of 25 μl, containing 2.5 μl buffer, 0.2 μm of 25 μM dNTPs, 0.5 μl of each 10 μM forward primer, 0.5 μm of the 10 μM reverse primer, 18.5 μl H_2_O and 0.25 μl 5 u/μl Taq polymerase. The PCR reaction was performed in a PTC-100 Cycler (MJ Research, Inc., Montreal, QC, Canada) under the following conditions: an initial denaturation step at 92 °C for 2 min 15 s, followed by 30 cycles of 92 °C for 15 s, 61 °C for 15 s, 72 °C for 30 s, and ending with a final elongation step at 72 °C for 1 min. Results were visualized on a 1 % agarose gel after 50 min electrophoresis at 110 V in 0.5× TAE (Tris-acetate-EDTA) buffer, using gel red staining at 1:20,000. The length of amplified products was used to determine the species of each sample. *C. obsoletus* exhibited products at 335 base pairs (bp), *C. scoticus* at 229 bp, *C. chiopterus* at 435 bp, and *C. dewulfi* at 493 bp.

### Morphometric measurements

Slide-mounted specimens were observed under a Nikon Alphaphot-2 YS2 compound light microscope (Nikon Instruments, Europe) with a Q Imaging (QI CAM) camera attachment, and measurements were taken using Image-Pro Plus software (Media Cybernetics Inc., Rockville, USA). Morphometric measurements were taken from the head, wings and abdomen of individuals. Fifteen variables were recorded and eight ratios were determined from the variables, based on measurements that appeared significant in terms of species discrimination in previous literature [26, 30, 31, 33].

### Head measurements

**Fig. 2:**
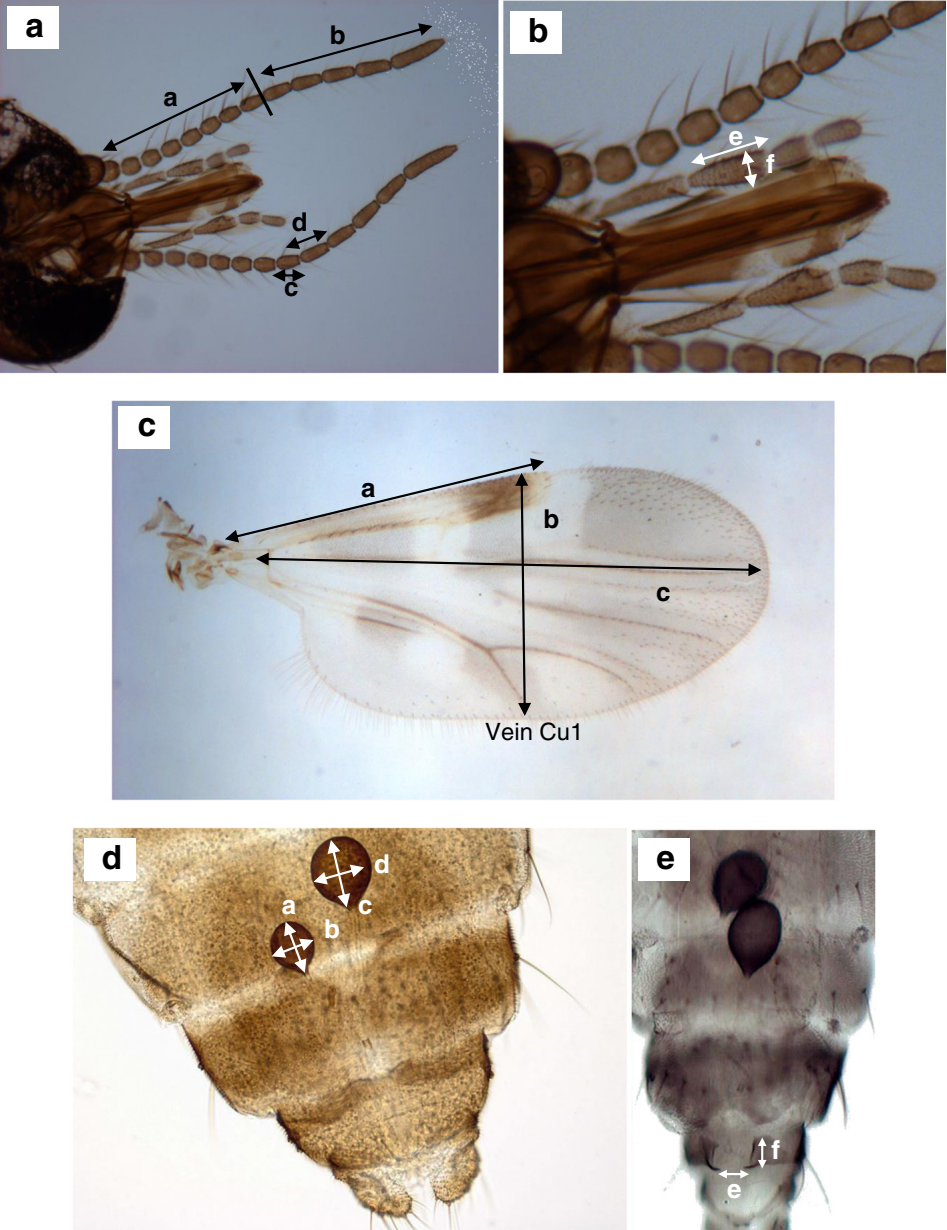
Morphometric measurements of *Culicoides*. **a** & **b** the head: where *a*) is length of eight basal flagellar segments of the antenna; *b*) is the length of the five apical flagellar segments of the antenna; *c*) is the length of flagellomere 10; *d*) is the length of flagellomere 11; *e*) is the length of the third palpal segment; and *f*) is the width of the third palpal segment. **c** the wing: where *a*) is the costa length; *b*) is the width of the wing; and *c*) is the length of the wing (arculus to tip). **d** & **e** the abdomen: where *a*) is the length of spermatheca 1; *b*) is the width of spermatheca 1; *c*) is the length of spermatheca 2; *d*) is the width of spermatheca 2; *e*) is the length between the chitinous plates; and *f*) is the width of the chitinous plates

From the maxillary palps, the length and width of the third palpal segment were measured, and the palpal ratio calculated (length: width) (Fig. 2b). From the antennae, the length of flagellomeres 10 and 11 were determined (Fig. 2a), as were the combined length of the five apical flagellar segments and eight basal flagellar segments. The antenna segment ratio [total length of 5 apical segments (11–15)/total length of 8 basal segments (3–10)] and flagella ratio (length of flagellomere 11/length of flagellomere 10) were calculated from the head measurements.

#### Wing measurements

The wing length from arculus to tip, costa length, and width of the wing (from the location of the second radial cell to the base of vein Cu1) were individually measured (Fig. 2c). These measurements were used to determine the wing ratio (wing length/width) and costa ratio (costa length/wing width).

#### Abdominal measurements

On the posterior part of the abdomen, the length and width of both spermathecae were measured along with the length and width between the chitinous plates surrounding the genital opening (Fig. 2d, e). The spermatheca ratio (spermatheca length/spermatheca width) was determined for both the larger and smaller spermathecae, and the chitinous plate ratio (length between chitinous plates/width of chitinous plates) was calculated using the abdominal measurements.

### Statistical analyses

Statistical differences between the measurements of *C. obsoletus* and *C. scoticus* taken in every region, between regions, and between seasons (for the samples from Calvados only), were determined using the non-parametric Mann-Whitney test (Anderson-Darling test for normality, *P* ≤ 0.05 for all measures). General Linear Models were used to assess the relative importance of both species and geographical location on the measurements and ratios, as well as the interaction of these two factors. An adjustment was made for multiple comparisons, using the Bonferroni correction, whereby the critical value for significance was adjusted to a lower threshold (e.g. if testing n hypotheses with a desired significance of 0.05, the Bonferroni correction would test the individual hypotheses at 0.05/n) [37].

Measurements of each variable were evaluated using the coefficient of variation (CV = SD/$\overline{X}$ ×100) and the coefficient of difference (CD = $\left({\overline{X}}_{\mathrm{A}}-{\overline{X}}_{\mathrm{B}}\right)$/(SD_A_ + SD_B_) for a given variable measured in two groups of individuals, A and B (where SD = standard deviation, and $\overline{X}$ = mean). CV describes the data heterogeneity whereas CD is linked to the degree of separation between two distributions. Mayr et al. consider 1.28 as the CD critical threshold at which members of one species can be considered different subspecies [38].

Principal component analysis (PCA) was performed in R 2.10.0 (R Development Core Team, 2009), and used to explore the correlation structure between variables and determine those variables that account for the greatest variance.

## Results

### Molecular analyses

A total of 819 *Culicoides* were identified to species level using the COI gene. These comprised 410 *C. obsoletus*, 348 *C. scoticus* and 61 *C. dewulfi/C. chiopterus*. For the French samples, five *C. obsoletus*, nine *C. scoticus* and two *C. dewulfi* were initially identified from a total of 91 samples from Landes. A second batch of samples from this location were then analysed to increase numbers, and from this batch 60 *C. obsoletus* and ten *C. scoticus* were identified from 98 specimens. None of the *C. dewulfi* or *C. chiopterus* samples identified molecularly were used to take morphometric measurements. See Table 1 for the total number of each species identified in each country and region.

**Table 1:** Total number of *C dewulfi*, *C. chiopterus*, *C. obsoletus* and *C. scoticus* identified using the cytochrome *c* oxidase subunit 1 (CO1) gene, from two regions in the UK, Spain and France

Country	Region	Species identified using COI gene				Total identified (total available)
		*C. obsoletus*	*C. scoticus*	*C. dewulfi*	*C. chiopterus*	
UK	Bala	48	69	9	0	126 (150)
	Devon	47	48	4	4	104 (106)
Spain	Avià	24	52	0	0	76 (77)
	Caldes de Malavella	56	40	1	0	97 (103)
France	Landes	65	19	2	0	86 (181)
	Calvados (start of season)	21	83	5	0	109 (115)
	Calvados (middle of season)	40	23	36	0	99 (124)
	Calvados (end of season)	109	14	0	0	123 (130)
Total		410	348	57	4	819 (986)

### Morphometric measurements

Morphometric measurements could not be undertaken on every sample that had been identified molecularly. Of 758 molecularly-identified *C. obsoletus* and *C. scoticus* individuals, four *C. obsoletus* and three *C. scoticus* from the UK had damaged flagellae, six *C. obsoletus* from Spain and four *C. scoticus* from France also had damaged flagellae, while one *C. obsoletus* from France had maxillary palp damage. The morphometric measurements for these *Culicoides* were not taken from the damaged structures, but all other measurements were collected.

The overall mean, standard deviation, CD and CV values of the *C. obsoletus* and *C. scoticus* measurements are shown in Table 2. CD values ranged from 0.02 (third palp length) to 4.30 (length of smaller spermatheca). Four variables (length and width of larger spermatheca, and length and width of smaller spermatheca) exhibited CD values over 1.28. None of these variables exhibited overlapping ranges when compared between the two species (*C. obsoletus* 44.17–51.97 μm, *C. scoticus* 57.03–65.98 μm; *C. obsoletus* 30–35.28 μm, *C. scoticus* 36.88–45.9 μm; *C. obsoletus* 44.06–49.98 μm, *C. scoticus* 56.19–64.99 μm; *C. obsoletus* 29–34.18 μm, *C. scoticus* 35.98–45.97 μm, respectively).

**Table 2:** Descriptive statistics, the coefficient of variation (CV) and Mayr’s coefficient of difference (CD) for 15 measurements and eight ratios of morphometric parameters in two *Culicoides* species (*C. obsoletus* and *C. scoticus*). The 758 *Culicoides* were trapped in the UK, France and Spain

Parameter	*C. obsoletus*		*C. scoticus*		CD
	Mean (SD)	CV	Mean (SD)	CV	
Wing length	1.27 (0.16)	12.26	1.34 (0.15)	11.32	0.22
Wing width	0.56 (0.26)	47.02	0.58 (0.06)	11.05	0.04
Costa length	0.80 (0.10)	12.8	0.83 (0.10)	12.36	0.15
Wing ratio	2.32 (0.15)	6.35	2.33 (0.06)	2.6	0.05
Costa ratio	1.59 (0.04)	2.23	1.61 (0.04)	2.38	0.35
Third palp length	51.94 (6.95)	13.39	51.67 (7.08)	13.7	0.02
Third palp width	21.47 (3.35)	15.61	17.87 (2.92)	16.32	0.57
Palpal ratio	2.46 (0.39)	15.68	2.96 (0.61)	20.42	0.51
Flagella 10 length	37.12 (3.61)	9.72	35.89 (3.72)	10.37	0.17
Flagella 11 length	49.32 (5.32)	10.78	50.33 (5.10)	10.12	0.10
5 Apical segment length	298.30 (26.52)	8.89	299.85 (25.36)	8.46	0.03
8 Basal segment length	271.94 (23.76)	8.74	259.02 (20.94)	8.08	0.29
Flagella ratio	1.10 (0.05)	4.62	1.16 (0.07)	6.24	0.50
Segment ratio	1.33 (0.11)	8.06	1.41 (0.14)	9.96	0.31
Larger spermatheca length	47.89 (1.74)	3.63	61.64 (2.18)	3.54	3.51
Larger spermatheca width	33.26 (1.47)	4.43	41.19 (2.48)	6.03	2.00
Smaller spermatheca length	47.29 (1.31)	2.77	61.03 (1.88)	3.09	4.30
Smaller spermatheca width	32.83 (1.59)	4.84	40.35 (2.80)	6.93	1.72
Larger spermatheca ratio	1.44 (0.07)	4.84	1.50 (0.10)	6.66	0.35
Smaller spermatheca ratio	1.44 (0.07)	4.87	1.52 (0.11)	7.37	0.42
Length between chitinous plates	11.91 (1.60)	13.41	20.25 (5.84)	28.85	1.12
Width of chitinous plates	18.31 (2.08)	11.37	20.68 (2.73)	13.18	0.49
Chitinous plate ratio	0.66 (0.10)	15.86	0.99 (0.31)	31.23	0.81

Units are expressed in μm, except for wing measurements, which are in mm

CV values for *C. obsoletus* ranged from 2.23 (costa ratio) to 47.02 (wing width), while for *C. scoticus* they ranged from 2.38 (costa ratio) to 31.23 (chitinous plate ratio). Clear differences can be seen in the means of the four characteristics that showed CD values greater than 1.28 (measurements all significantly larger for *C. scoticus* than for *C. obsoletus*, Mann-Whitney test, *P* ≤ 0.0001).

The Pearson’s *r* correlation matrix of the 15 morphometric characteristics, used for the primary multivariate analyses, exhibited a high degree of correlation, even when the *P* value was modified following Bonferroni correction (*P* ≤ 0.0004) (Additional file 1). Most pairs of variables exhibited positive correlation, with the strongest relationship between the spermathecae measurements (*r* > 0.80). Almost all of the ratios displayed significant levels of positive correlation (*P* ≤ 0.002), although this relationship was weaker than for the morphometric measurements (maximum *r-*value = 0.458).

#### Differentiation between C. obsoletus and C. scoticus within each country

Statistical differences in morphometric measurements (Table 3) and ratios (Table 4) between *C. obsoletus* and *C. scoticus* were determined for each geographical region. The head measurements were the least able to differentiate between the two species in any location, with the length of flagellomere 11, and the length of the five apical segments only significantly different between the species at the end of the season in France. The width of the third segment of the maxillary palp, however, was statistically different between the species in all locations. The abdominal measurements also exhibited significant interspecific differences in every region. In contrast, for the morphometric ratios, the wing ratios did not enable consistent differentiation between the two species while those based on head measurements showed significant inter-specific differences at all locations.

**Table 3:** A summary of 15 morphological measurements of *C. obsoletus* and *C. scoticus* that exhibited significant differences between the species from sites in the UK, France and Spain

Measurement	UK		France				Spain	
	Bala	Devon	Landes	Calvados			Avià	Caldes
				Start	Middle	End		
Wing length		×	×	×		×	×	×
Wing width		×	×			×	×	
Costa length		×	×			×		
Third palp length			×			×		
Third palp width	×	×	×	×	×	×	×	×
Length of flagella 10	×	×	×					
Length of flagella 11						×		
5 Apical segment length						×		
8 Basal segment length	×	×	×		×		×	×
Larger spermatheca length	×	×	×	×	×	×	×	×
Larger spermatheca width	×	×	×	×	×	×	×	×
Smaller spermatheca length	×	×	×	×	×	×	×	×
Smaller spermatheca width	×	×	×	×	×	×	×	×
Chitinous plate length	×	×	×	×	×	×	×	×
Chitinous plate width	×	×	×	×	×	×	×	×

× indicates a significant difference between the means

**Table 4:** A summary of eight ratios derived from morphological measurements of *C. obsoletus* and *C. scoticus* that exhibited significant differences between the species from sites in the UK, France and Spain

Ratio	UK		France				Spain	
	Bala	Devon	Landes	Calvados			Avià	Caldes
				Start	Middle	End		
Wing ratio		×						
Costa ratio	×	×			×		×	×
Palp ratio	×	×	×	×	×	×	×	×
Segment ratio	×	×	×	×	×	×	×	×
Flagella ratio	×	×	×	×	×	×	×	×
Larger spermatheca ratio	×	×	×	×	×		×	×
Smaller spermatheca ratio	×	×	×	×	×		×	×
Chitinous plate ratio	×	×	×	×	×	×	×	×

× indicates a significant difference between the means within each region

#### Comparisons of C. obsoletus and C. scoticus between countries and regions

Tables containing full results of the statistical tests can be found in Additional file 1, and are summarised below. For comparisons, significance was determined, using the Bonferroni correction, where *P ≤* 0.002.

##### Head

For *C. obsoletus*, the length and width of the third segment of the maxillary palp, the length of flagellomeres 10 and 11, as well as the length of the five apical and eight basal segments, were significantly longer in *Culicoides* from Landes compared to the other locations tested. The palpal ratio was smallest for the Spanish sites. The flagella and segment ratios were not significantly different between the sites.

For *C. scoticus* the length of the third palpal segment was significantly smaller for the two Spanish sites than the collections from Landes or Bala. The width of the third palpal segment of individuals from Devon was significantly smaller than in all other collections. Flagellomere 10 was significantly smaller in collections from Devon and Caldes de Malavella, when compared to those from Bala and France. Flagellomere 11, and the five apical and eight basal segments were significantly larger in Landes than any other region. The palpal ratio of the Spanish collections was significantly smaller than those from Bala and Calvados. For the flagella ratio, the collections from Avià were significantly larger than those from the two UK sites or Calvados. There were no significant differences in the segment ratio between the regions.

The interaction between species and location was only significant for the flagella ratio. The fitted means from the GLM for the factors ‘species’ and ‘location’, as well as their interaction, can be found in Additional file 1.

##### Wings

For *C. obsoletus*, the wing length and costa length for samples from Caldes de Malavella were significantly smaller than for samples from other sites. These measurements for Landes were significantly larger than the other sites. The costa ratio was smallest for Bala and Calvados, while the wing ratio was not significantly different between sites.

For *C. scoticus* the wing length, width, and costa length was significantly smaller for Caldes de Malavella than the other sites. There were no significant differences in wing ratio between the sites, but the costa ratio was significantly higher for the two Spanish sites than France or Bala.

The interaction between species and location was only significant for wing length and costa length (Additional file 1).

##### Abdomen

For *C. obsoletus*, the length of the larger spermatheca was significantly longer in samples from Landes than from the other regions (overall range 44–52 μm). The length of the smaller spermatheca was significantly different for the *Culicoides* from Spain, when compared to those from the UK or France. The length between the chitinous plates was significantly shorter for the Spanish samples than for the French samples. The width of the chitinous plates was significantly longer for Landes than for the other sites. There were no significant differences in the larger spermatheca or chitinous plate ratios, but the smaller spermatheca ratio was significantly smaller in samples from France than for samples from Caldes de Malavella.

For *C. scoticus*, the length of the larger spermatheca was significantly smaller in samples from Caldes de Malavella than France (overall range 57–66 μm). The width of the larger spermatheca was significantly smaller in midges from Spain. The smaller spermatheca width and width between the chitinous plates was significantly shorter in samples from Spain than those from France or Bala. The chitinous plate length and ratio, as well as both the larger and smaller spermatheca ratios did not differ significantly between sites.

The interaction between species and location was not significant for any of the abdominal measurements or ratios (Additional file 1).

#### *Comparisons between seasons in Calvados, France*

##### Head

*Culicoides obsoletus* exhibited no significant difference in third palpal segment width, or in the length of flagellomeres 10 and 11, between the start, middle and end of the season. The length of the five apical segments was greater at the start than in the middle of the season.

In *C. scoticus* flagellomeres 10 and 11, the five apical segments and the eight basal segments of the antenna were all significantly smaller in collections from the middle of the season compared to the start and end of the season.

##### Wings

In *C. obsoletus*, the wing length for the middle of the season was significantly smaller than that at the start and end of the season. The wing width and costa length for the start, middle and end of the season were all significantly different from each other. The wing ratio was significantly larger for the middle of the season compared to the start.

For *C. scoticus* the costa length in the middle of the season was significantly smaller than at the start and end.

##### Abdomen

For both *C. obsoletus* and *C. scoticus*, there was no difference between seasons in the abdominal measurements.

#### *Principal component analyses*

##### Morphometric measurements

**Fig. 3:**
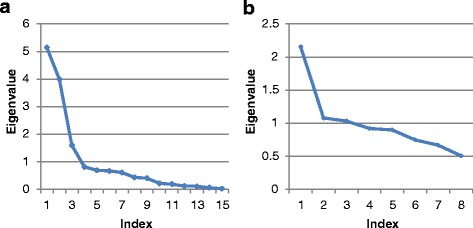
A scree plot highlighting the relationship between the eigenvalues and the number of axis in a principal component analysis of **a** 15 morphometric measurements of *C. obsoletus* and *C. scoticus*; and **b** eight ratios derived from morphometric measurements of those individuals

Morphometric differences were studied through PCA on the measurement data. Kaiser’s stopping rule states that only the number of axes with eigenvalues over 1.00 should be considered in the analysis. The initial analysis of the 15 morphometric measurements indicated that three axes had an eigenvalue of 1.00 or higher (Table 5) and when combined, these factors accounted for 71.5 % of the variance. The scree plot (Fig. 3a) confirms the relationship between the relative magnitude of the eigenvalues and the number of axis [39].

**Table 5:** The eigenvalues, percent variance and cumulative variance of the axes from the principal component analysis of 15 morphometric measurements of *C. obsoletus* and *C. scoticus*

Axis (principal component)	Initial eigenvalues		
	Total	% of Variance	Cumulative %
1	5.142	34.283	34.283
2	3.991	26.608	60.891
3	1.592	10.610	71.501
4	0.812	5.412	76.913
5	0.688	4.585	81.498
6	0.664	4.424	85.921
7	0.603	4.020	89.941
8	0.433	2.886	92.827
9	0.399	2.657	95.484
10	0.212	1.411	96.895
11	0.182	1.213	98.108
12	0.114	0.758	98.866
13	0.102	0.683	99.548
14	0.054	0.359	99.907
15	0.014	0.093	100.000

**Fig. 4:**
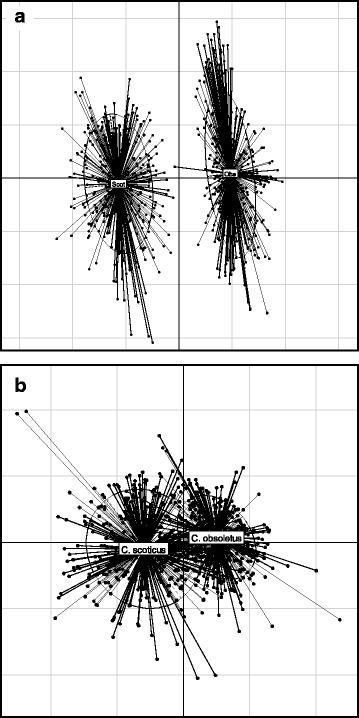
Results of principal component analysis on **a** morphological measurements of *C. obsoletus* and *C. scoticus*; and **b** morphometric ratios of *C. obsoletus* and *C. scoticus*

The PCA scatterplot unambiguously separated *C. obsoletus* and *C. scoticus* (Fig. 4a). The first axis (X axis; PC1) was highly negatively correlated to the lengths and widths of the larger and smaller spermathecae (loadings ≥ 0.8), as well as the length between, and width of, the chitinous plates (loadings ≥ 0.55), and fairly correlated to wing length, costa length and third palpal segment width (loadings ≥ 0.45). The second axis (Y axis; PC2) was positively correlated with antennal segment lengths (Table 6).

**Table 6:** Characterisation of *C. obsoletus* and *C. scoticus* using the loadings of principal component analyses on 15 morphometric parameters

Parameter	Principal component		
	PC1	PC2	PC3
Larger spermatheca length	**-0.914**	-0.212	-0.190
Larger spermatheca width	-**0.895**	-0.158	-0.146
Smaller spermatheca length	-**0.913**	-0.215	-0.202
Smaller spermatheca width	-**0.870**	-0.155	-0.133
Length between chitinous plates	**-0.767**	-0.163	-0.083
Width of chitinous plates	**-0.600**	0.019	0.056
Third palp length	-0.168	**0.600**	-0.150
Third palp width	**0.476**	**0.483**	-0.012
Flagella 10 length	-0.032	**0.812**	-0.209
Flagella 11 length	-0.310	**0.787**	-0.259
5 Apical segment length	-0.248	**0.866**	-0.252
8 Basal segment length	0.030	**0.910**	-0.123
Wing length	**-0.539**	**0.414**	**0.669**
Wing width	-0.180	0.148	**0.556**
Costa length	-**0.481**	**0.424**	**0.705**

PC1, first axis; PC2, second axis; PC3, third axis. Loadings of fair correlation, or above, are highlighted in bold

##### Morphometric ratios

The eight morphometric ratios were also subjected to PCA, to examine differences in shape between *C. obsoletus* and *C. scoticus*. Kaiser’s stopping rule suggested the inclusion of the first three axes (Table 7), while the scree test (Fig. 3b) suggested inclusion of only the first axis. The structure of the data was also weak, with seven of the eight axes accounting for similar amounts of variance each (6–13 %). This was confirmed by a scatterplot of the first three axes, which was unable to separate the two species (Fig. 4b). PC1 was negatively correlated to the majority of ratios (Table 8).

**Table 7:** The eigenvalues, percent variance and cumulative variance of the axes from the principal component analysis of eight ratios derived from morphometric measurements of *C. obsoletus* and *C. scoticus*

Factor	Initial eigenvalues		
	Total	% of Variance	Cumulative %
1	2.154	26.930	26.930
2	1.078	13.479	40.409
3	1.031	12.889	53.299
4	0.919	11.482	64.781
5	0.897	11.210	75.991
6	0.747	9.337	85.328
7	0.666	8.323	93.651
8	0.508	6.349	100.000

**Table 8:** Characterisation of *C. obsoletus* and *C. scoticus* using the loadings of principal components analyses on eight ratios derived from the morphometric parameters

Parameter	Principal component		
	PCA1	PCA2	PCA3
Larger spermatheca ratio	**-0.457**	-0.057	-0.048
Smaller spermatheca ratio	**-0.488**	**-0.533**	-0.008
Chitinous plate ratio	**-0.652**	-0.227	0.122
Palpal ratio	**-0.502**	-0.262	0.234
Flagella ratio	**-0.707**	**0.425**	0.054
Segment ratio	**-0.564**	**0.636**	0.142
Wing ratio	-0.105	0.161	**-0.861**
Costa ratio	**0.450**	-0.244	**-0.442**

PC1, first axis; PC2, second axis; PC3, third axis. Loadings of fair correlation, or above, are highlighted in bold

## Discussion

This study is the first to investigate whether traditional morphological or morphometric identification techniques can be applied to female *C. obsoletus* and *C. scoticus* individuals trapped at different geographical regions and at different time periods during the vector season. The results demonstrate that abdominal measurements could be used to reliably separate the two species irrespective of trapping location or time of year. The implication is that by using this technique to differentiate between the species, both time and money could be saved by not undertaking molecular identification methods.

The aim of this study was to assess existing methods for differentiating *C. obsoletus* and *C. scoticus* using a range of morphological and morphometric techniques [26, 30, 31, 33]. The study examined the likelihood of error arising from basing such studies on a limited geographic or temporal range of samples and specimens identified to species level using a multiplex PCR assay based on the COI mitochondrial gene. Geographical and seasonal variation in size was demonstrated in both species, with *Culicoides* exhibiting smaller measurements being found at more southern latitudes (e.g. *Culicoides* from Caldes de Malavella exhibited smaller measurements than those from other areas, with the exception of measurements for the third palpal segment width). However, abdominal measurements could be used to reliably separate *C. obsoletus* and *C. scoticus* even taking into account this variation. While it was found that the length and width of the spermathecae can be used to consistently differentiate between *C. obsoletus* and *C. scoticus*, this can be a time-consuming process.

A major advance in the current study was the use of a relatively large number of samples and individuals from a wide geographical and temporal range. The demonstration that these factors were important in determining the size of individuals examined had not been accounted for in previous studies of traditional morphometrics at smaller scales [31, 35]. This has consequences in cases where quantitative measurements are used for separation and preliminary trials establishing local variation in size within the region and time of interest should be considered.

A study carried out in Sweden and Denmark had previously suggested that it is possible to separate the females of the four species by combining the shape of the third segment of the maxillary palp [30], whereby the palpal ratio for *C. obsoletus* < 2.6 and *C. scoticus* > 2.7. In the current study however, although we identified similar mean values to those identified by Nielsen & Kristensen [30] (2.5 for *C. obsoletus* and 2.9 for *C. scoticus*), the range of these values overlapped between species and could not be used to differentiate between them. The *Culicoides* used for their identifications however, were all collected from Sweden or Denmark, so did not take into account the possible influence of geographical differences in *Culicoides* morphology, or the effect of seasonal variation (all samples were collected between July and September).

Correlation was observed between the morphometric measurements, as well as the ratios derived from the morphometric characteristics. This was particularly true in the case of spermatheca lengths and widths, whereby strong positive correlations were observed between these measurements as well as between the larger and smaller spermathecae. A multivariate PCA was undertaken to transform the correlated variables into a smaller number of linearly uncorrelated variables (principal components) to explain the variability in the data. The current study confirmed that *C. obsoletus* and *C. scoticus* can be separated by the length and width of the larger and smaller spermatheca [31]. The lack of diagnostic value in the morphometric ratios, as determined by the weak data structure and overlapping scatter-plots of a PCA on these data, also confirmed this previous study. Although it is possible to distinguish between these two species using a scheme based on four characteristics, these are time-consuming to measure and may require the use of slide mounting individuals, as it is not always easy to obtain direct access to the spermathecae of individuals with their abdomens attached.

In a study by Muñoz-Muñoz [34], it was suggested that changes in wing shape between distant conspecific populations of *Culicoides* are not a result of size variation, but that genetic differences may arise as species-specific adaption to particular environments. When comparing geographical differences in the size of the two species in the current study however, the *Culicoides* collected in Spain were smaller than those in samples originating from other locations, with significantly smaller palps, flagellae, wings, spermathecae and chitinous plates. This was particularly the case in samples collected in Caldes de Malavella and highlights the importance of taking into account regional variation in morphological/morphometric measurements when identifying *Culicoides*.

An inverse relationship has been established previously between wing length and larval rearing temperatures both in the laboratory [40] and in the field [41]. Seasonal variation was observed in the morphometric measurements from the *Culicoides* trapped in Calvados. All flagellomere measurements were significantly smaller in the middle of the season than at the start and the end of the season. The same trend was seen for wing and costa length. These seasonal variations are likely to be due to variation in temperature between these time-points, with the peak in temperature exhibited at the middle of the season, and therefore smaller measurements occurring at this time than at the start and end of the season, when the temperatures would be cooler. The abdominal measurements, however, did not show any significant differences between seasons, highlighting the reliability of using abdominal morphometrics to discriminate between the species.

Four variables (the length and width of the larger and smaller spermathecae) exhibited CD values greater than 1.28 and the means of these variables did not overlap, indicating that these characteristics can be used to distinguish between species [31]. None of the ratios exhibited values above the critical threshold. The lack of overlap between spermatheca length was also highlighted by Delécolle, who concluded that all females with a larger spermatheca length ≤ 59 μm were *C. obsoletus* and those with a larger spermatheca length were *C. scoticus* (overall range of measurements 50–67 μm) [26]. In this study, specimens with larger spermatheca lengths ≤ 52 μm were *C. obsoletus* and ≥ 57 μm were *C. scoticus* (overall range 44–66 μm). Since there is a gap of 5 μm between larger spermatheca length in the *C. obsoletus* and *C. scoticus* individuals measured here, it would be advisable to molecularly identify any individual that falls within this range 52 to 57 μm. The same is true for the gap seen between the species measurements for the smaller spermatheca length, and less so for the larger and smaller spermathecae widths.

There are a number of PCR-based methods currently available to differentiate between the two species. While molecular methods have their own relative benefits, such as clear objectivity in comparison to morphological interpretation, molecular techniques are more expensive than microscope-based identifications. As *Culicoides* are manually sorted into species groups prior to molecular identification, often by the people who go on to do the PCR work, it may well save time and costs to undertake morphometric identification on a subsample of individuals to determine *C. obsoletus* and *C. scoticus* prevalence.

This work is a re-evaluation of morphometric variables previously assessed by Delécolle [26], Pagès & Sarto I Monteys [33], Augot et al. [31] and Nielsen & Christensen [30] in discriminating between *C. obsoletus* and *C. scoticus*. Of those studies, the work by Augot et al. was the most comprehensive in its statistical undertakings and comparison of morphometric features, and also enabled ‘sets’ of discriminatory features to be assessed. In order for this study to be comparable to the previous studies we undertook a number of the same statistical analyses (e.g. CD, CV, PCA). In addition to those analyses we used the non-parametric Mann-Whitney test to determine differences between *C. obsoletus* and *C. scoticus* measurements in every region, between regions and between seasons, and also used GLMs to assess the relative importance of both species and geographical location on the measurements and ratios (and their interaction). Together, these analyses provide a thorough comparison of measured morphometric variables.

Despite the similarities in the analyses undertaken, there are a number of differences between the studies. Augot et al. [31] collected 92 females *Culicoides* from two sites in France and identified 64 *C. obsoletus* and 18 *C. scoticus* (82 individuals) in total. Here, we collected 994 *Culicoides* from two sites in each of the UK, France and Spain and identified 410 *C. obsoletus* and 348 *C. scoticus* (758 individuals) in total.

Augot et al. [31] collected *Culicoides* between July and September and did not take into account seasonal variation; here we collected *Culicoides* from three points during the year (April, July and November) in France. This study uniquely highlights that both head and wing morphometrics differ seasonally, while abdominal measurements are not influenced by seasonality.

While both studies targeted the COI gene, Augot et al. [31] used phlebotomine sandfly (Diptera: Psychodidae) primers to sequence their samples and used two known males of *Culicoides* to identify whether their sequences were *C. obsoletus* or *C. scoticus*. Here, we used *Culicoides*-specific primers (Nolan et al. [10]) for the four *Avaritia* species (*C. obsoletus*, *C. scoticus*, *C. chiopterus* and *C. dewulfi*) - enabling us to determine the species of each sample based on the length of amplified products. This precluded us from investigating intraspecific differences based on COI, or divergence among species. But Augot et al. [31] concluded that they found no evidence of intraspecific differences in their samples.

We re-examined 13 of 15 measurements that Augot et al. [31] explored (excluding the length of the joint between both eyes and the area of the triangle defined by the three sensilla above the eyes due to the high CV and low CD values). We added two extra measurements, the total length of the 5 apical segments, and the total length of the 8 basal segments of the antenna. These additional measurements were used by Nielsen & Kristensen [30] to produce an ‘antenna ratio’ which they stated differentiated significantly between *C. obsoletus* and *C. scoticus*. We re-examined the seven ratios used by Augot et al. along with Nielsen & Kristensen’s ratio, yet none were able to discriminate between the species.

Augot et al. [31] concluded that *C. obsoletus* and *C. scoticus* can be determined using multivariate analyses based on the length & width of spermatheca 1, the length of spermatheca 2, and the width between the chitinous plates. Our results, however, highlight that the following individual morphological discontinuities can be used to differentiate *C. obsoletus* and *C*. scoticus:

- **Larger spermatheca length**: ≤ 52 μm *C. obsoletus* (range 44.17–51.97 μm); ≥ 57 μm *C. scoticus* (range 57.03–65.98 μm);
- **Smaller spermatheca length**: ≤ 50 μm *C. obsoletus* (range 44.06–49.98 μm); ≥ 56 μm *C. scoticus* (range 56.19–64.99 μm);
- **Larger spermatheca width**: ≤ 35.3 μm *C. obsoletus* (range 30–35.28 μm); ≥ 36.9 μm *C. scoticus* (range 36.88–45.9 μm);
- **Smaller spermatheca width**: ≤ 34.2 μm *C. obsoletus* (range 29–34.18 μm); ≥ 36 μm *C. scoticus* (range 35.98–45.97 μm).

## Conclusions

In conclusion, we have shown that female *C. obsoletus* and *C. scoticus* individuals can be separated under a stereomicroscope based on abdominal measurements. Seasonal variation in the size of these species, and therefore their morphometric measurements was observed for both head and wing measurements, but not for the abdomen. Our results confirm those concluded in a previous study by Augot et al. [31], while also taking into account geographical variation in the size of individual *Culicoides*. This observation is likely to be related to temperature at the trapping sites. Although the length and width of the spermathecae can be used to differentiate between the species, this can be a time-consuming process and we therefore recommend undertaking this on a subsample of individuals.

## Additional file

Additional file 1: Correlation matrices of 15 morphometric characteristics, and 8 ratios derived from these characteristics, for *C. obsoletus* and *C. scoticus*. (DOCX 43 kb)

## Abbreviations

BTV: bluetongue virus
BTV-8: bluetongue virus serotype 8
CD: coefficient of difference
CDC: Centers for Disease Control (with reference to an LED trap)
COI: cytochrome *c* oxidase subunit I
CV: coefficient of variation
ITS-1 (2): internal transcribed spacer 1 (or 2)
OVI: Onderstepoort Veterinary Institute (down-draught black light traps)
PC1: the first principal component
PC2: the second principle component
PCA: principal component analysis
SBV: Schmallenberg virus
TAE: Tris-acetate-EDTA buffer
