# Supplementary material for: Morphometric discrimination of two sympatric sibling species in the Palaearctic region, Culicoides obsoletus Meigen and C. scoticus Downes & Kettle (Diptera: Ceratopogonidae), vectors of bluetongue and Schmallenberg viruses
Source: Parasit Vectors. 2016 May 4;9:262. doi: 10.1186/s13071-016-1520-7 (PMC4857416; doi:10.1186/s13071-016-1520-7)
Supplement: Additional file 1: — Correlation matrices of 15 morphometric characteristics, and 8 ratios derived from these characteristics, for C. obsoletus and C. scoticus. (DOCX 43 kb) [file 13071_2016_1520_MOESM1_ESM.docx]

**Additional Table 1.** Correlation matrix of 15 morphometric characteristics of *C. obsoletus* and *C. scoticus*

| Larger spermatheca  Width | | 0.881* |  |  |  |  |  |  |  |  |  |  |  |  |  |
| --- | --- | --- | --- | --- | --- | --- | --- | --- | --- | --- | --- | --- | --- | --- | --- |
| Smaller spermatheca | Length | 0.945* | 0.886* |  |  |  |  |  |  |  |  |  |  |  |  |
|  | Width | 0.849* | 0.786* | 0.856* |  |  |  |  |  |  |  |  |  |  |  |
| Chitinous Plate | Length between | 0.71* | 0.678* | 0.701* | 0.614* |  |  |  |  |  |  |  |  |  |  |
|  | Width | 0.462* | 0.472* | 0.461* | 0.442* | 0.387* |  |  |  |  |  |  |  |  |  |
| 3^rd^ Palp | Length | 0.056 | 0.068 | 0.058 | 0.061 | 0.029 | 0.09 |  |  |  |  |  |  |  |  |
|  | Width | -0.479* | -0.435* | -0.486* | -0.427* | -0.371* | -0.247* | 0.197* |  |  |  |  |  |  |  |
| Flagella Segments | 10 Length | -0.102 | -0.063 | -0.1 | -0.072 | -0.082 | 0.023 | 0.426* | 0.289* |  |  |  |  |  |  |
|  | 11 Length | 0.155* | 0.163* | 0.15* | 0.17* | 0.113 | 0.179* | 0.45* | 0.196* | 0.589* |  |  |  |  |  |
|  | 5Apical Length | 0.086 | 0.112 | 0.091 | 0.11 | 0.069 | 0.113 | 0.49* | 0.27* | 0.701* | 0.869* |  |  |  |  |
|  | 8Basal Length | -0.2* | -0.14* | -0.205* | -0.151* | -0.153* | 0.006 | 0.472* | 0.389* | 0.774* | 0.687* | 0.797* |  |  |  |
| Wing | length | 0.281* | 0.32* | 0.269* | 0.313* | 0.279* | 0.308* | 0.223* | -0.064 | 0.207* | 0.315* | 0.315* | 0.262* |  |  |
|  | width | 0.062 | 0.078 | 0.06 | 0.082 | 0.078 | 0.105 | 0.079 | -0.004 | 0.023 | 0.053 | 0.072 | 0.08 | 0.314* |  |
| Costa  Length | | 0.212* | 0.257* | 0.201* | 0.26* | 0.237* | 0.284* | 0.207* | -0.045 | 0.214* | 0.296* | 0.302* | 0.271* | 0.983* | 0.318* |
|  | | Larger Spermatheca Length | Larger Spermatheca Width | Smaller Spermatheca Length | Smaller Spermatheca Width | Length between Chitinous Plates | Chitinous Plate Width | 3^rd^ Palp Length | 3^rd^ Palp Width | Flagella 10 Length | Flagella 11 Length | 5 Apical Segment Length | 8 Basal Segment Length | Wing length | Wing width |

Significance, determined using the Bonferroni correction, is given where **P ≤ 0.0004*

**Additional Table 2.** Correlation matrix of 8 ratios derived from morphometric characteristics of *C. obsoletus* and *C. scoticus*

|  | Larger Spermatheca Ratio | Smaller Spermatheca Ratio | Chitinous Plate Ratio | Palpal Ratio | Flagella Ratio | Segment Ratio | Wing Ratio |
| --- | --- | --- | --- | --- | --- | --- | --- |
| Smaller Spermatheca Ratio | 0.083 |  |  |  |  |  |  |
| Chitinous Plate Ratio | 0.193* | 0.271* |  |  |  |  |  |
| Palpal Ratio | 0.161* | 0.171* | 0.274* |  |  |  |  |
| Flagella Ratio | 0.199* | 0.184* | 0.311* | 0.179* |  |  |  |
| Segment Ratio | 0.119* | 0.068 | 0.177* | 0.136* | 0.458* |  |  |
| Wing Ratio | 0.023 | -0.005 | 0.023 | 0.008 | 0.066 | 0.017 |  |
| Costa Ratio | 0.157* | 0.21* | 0.16* | 0.089 | 0.171* | 0.119* | 0.082 |

Significance determined using the Bonferroni correction, is given where **P ≤ 0.002*

**Additional Table 3.** The fitted means resulting from a general linear model to assess the relative role of ‘species’ and ‘locality’ on 15 morphometric measurements of *C. obsoletus* and *C. scoticus*, along with eight ratios derived from the morphometric measurements.

| **Measurement** | **Factor** | | | | | | | | | |
| --- | --- | --- | --- | --- | --- | --- | --- | --- | --- | --- |
|  | **Species** | | | **Location** | | | | | | |
|  | ***C. obsoletus*** | ***C. scoticus*** | ***P*-Value** | **Bala** | **Devon** | **Calvados** | **Landes** | **Avià** | **Caldes de Malavella** | ***P*-Value** |
| Wing Length* | 1.27 | 1.32 | 0.142 | 1.37 | 1.30 | 1.34 | 1.41 | 1.26 | 1.08 | 0.016 |
| Wing Width | 0.56 | 0.58 | 0.273 | 0.63 | 0.56 | 0.57 | 0.63 | 0.54 | 0.47 | 0.000 |
| Costa Length* | 0.80 | 0.82 | 0.288 | 0.86 | 0.81 | 0.84 | 0.88 | 0.78 | 0.66 | 0.009 |
| Wing Ratio | 2.32 | 2.33 | 0.281 | 2.32 | 2.31 | 2.34 | 2.31 | 2.33 | 2.30 | 0.049 |
| Costa Ratio | 1.59 | 1.61 | 0.000 | 1.59 | 1.60 | 1.59 | 1.60 | 1.62 | 1.63 | 0.000 |
|  |  |  |  |  |  |  |  |  |  |  |
| 3^rd^ Palp Length | 51.85 | 52.19 | 0.468 | 52.97 | 51.58 | 50.94 | 59.26 | 48.92 | 48.43 | 0.000 |
| 3^rd^ Palp Width | 21.71 | 18.23 | 0.000 | 19.93 | 17.72 | 18.94 | 22.38 | 20.81 | 20.05 | 0.000 |
| Palpal Ratio | 2.43 | 2.95 | 0.000 | 2.75 | 3.03 | 2.73 | 2.72 | 2.41 | 2.50 | 0.000 |
| Flagella 10 Length | 36.95 | 36.04 | 0.000 | 37.00 | 35.36 | 36.44 | 39.95 | 35.55 | 34.66 | 0.000 |
| Flagella 11 Length | 49.42 | 50.94 | 0.000 | 50.46 | 49.57 | 48.78 | 56.47 | 48.73 | 47.07 | 0.000 |
| 5 Apical Segment Length | 298.57 | 303.09 | 0.008 | 301.39 | 295.16 | 294.29 | 333.26 | 297.73 | 287.14 | 0.000 |
| 8 Basal Segment Length | 271.07 | 261.28 | 0.000 | 268.28 | 262.59 | 262.62 | 295.66 | 253.84 | 254.10 | 0.000 |
| Flagella Ratio* | 1.10 | 1.16 | 0.001 | 1.13 | 1.13 | 1.12 | 1.13 | 1.15 | 1.14 | 0.593 |
| Segment Ratio | 1.34 | 1.42 | 0.000 | 1.37 | 1.41 | 1.35 | 1.42 | 1.37 | 1.36 | 0.000 |
|  |  |  |  |  |  |  |  |  |  |  |
| Larger Spermatheca Length | 47.63 | 61.60 | 0.000 | 54.92 | 54.20 | 55.21 | 56.07 | 53.84 | 53.44 | 0.000 |
| Larger Spermatheca Width | 32.97 | 41.05 | 0.000 | 37.57 | 36.86 | 38.79 | 38.02 | 36.28 | 35.56 | 0.000 |
| Smaller Spermatheca Length | 47.03 | 60.89 | 0.000 | 54.36 | 53.83 | 54.67 | 54.67 | 53.35 | 52.89 | 0.000 |
| Smaller Spermatheca Width | 32.55 | 40.26 | 0.000 | 36.98 | 36.13 | 37.09 | 37.65 | 35.53 | 35.00 | 0.000 |
| Larger Spermatheca Ratio | 1.45 | 1.51 | 0.000 | 1.46 | 1.47 | 1.46 | 1.47 | 1.49 | 1.50 | 0.001 |
| Smaller Spermatheca Ratio | 1.45 | 1.52 | 0.000 | 1.47 | 1.49 | 1.47 | 1.45 | 1.50 | 1.51 | 0.000 |
|  |  |  |  |  |  |  |  |  |  |  |
| Length between Chitinous Plates | 11.58 | 20.12 | 0.000 | 16.02 | 15.54 | 16.77 | 17.03 | 15.31 | 14.41 | 0.000 |
| Width of Chitinous Plates | 18.09 | 20.66 | 0.000 | 20.00 | 18.95 | 19.84 | 21.12 | 18.68 | 17.63 | 0.000 |
| Chitinous Plate Ratio | 0.65 | 0.99 | 0.000 | 0.80 | 0.82 | 0.84 | 0.81 | 0.83 | 0.82 | 0.468 |

Significance, determined using the Bonferroni correction, should be considered where *P ≤ 0.002*.

* indicates a significant interaction of ‘species’ and ‘location’ – results of those interactions can be seen in Additional Table 4 .

**Additional Table 4.** The fitted means resulting from a general linear model to assess the interaction of ‘species’ and ‘locality’ on 15 morphometric measurements of *C. obsoletus* and *C. scoticus*, along with eight ratios derived from the morphometric measurements.

| **Measurement** | **Species** | **Location** | | | | | | |
| --- | --- | --- | --- | --- | --- | --- | --- | --- |
|  |  | **Bala** | **Devon** | **Calvados** | **Landes** | **Avià** | **Caldes de Malavella** | ***P*-Value** |
| Wing Length | Obsoletus | 1.36 | 1.24 | 1.27 | 1.46 | 1.23 | 1.04 | 0.000 |
|  | Scoticus | 1.39 | 1.36 | 1.41 | 1.37 | 1.28 | 1.11 |  |
|  |  |  |  |  |  |  |  |  |
| Costa Length | Obsoletus | 0.86 | 0.78 | 0.81 | 0.91 | 0.77 | 0.65 | 0.000 |
|  | Scoticus | 0.87 | 0.84 | 0.88 | 0.86 | 0.79 | 0.67 |  |
|  |  |  |  |  |  |  |  |  |
| Flagella Ratio | Obsoletus | 1.12 | 1.10 | 1.09 | 1.10 | 1.10 | 1.09 | 0.002 |
|  | Scoticus | 1.15 | 1.15 | 1.15 | 1.16 | 1.20 | 1.18 |  |

Significance, determined using the Bonferroni correction, should be considered where *P ≤ 0.002*.

The interaction plots highlight that the mean wing and costa length for *C. scoticus* is consistently greater than that for *C. obsoletus* in all locations except for Landes, where these measures are greater for *C. obsoletus*. For the flagella ratio, this measurement is consistently greater for *C. scoticus* in all locations, yet the difference between the means is less in Bala and Devon, and greatest in Caldes de Malavella and Avià.

**Additional Table 5.** A summary of 15 morphological measurements and eight ratios of *C. obsoletus* that exhibited significant differences between sites in the UK, France and Spain.

| **Measurement** | **UK** | | | **France** | | | **Spain** | | |
| --- | --- | --- | --- | --- | --- | --- | --- | --- | --- |
|  | **Bala** | | **Devon** | **Landes** | **Calvados** | | **Avià** | **Caldes de Malavella** | |
| Wing Length | 1.36C | 1.24B | | 1.46D | | 1.27B | 1.23B | | 1.04A |
| Wing Width | 0.69C | 0.54ABC | | 0.63BC | | 0.54AB | 0.53ABC | | 0.47A |
| Costa Length | 0.86C | 0.78B | | 0.91D | | 0.81B | 0.77B | | 0.65A |
| Wing Ratio* | 2.29A | 2.30A | | 2.31A | | 2.34A | 2.34A | | 2.29A |
| Costa Ratio | 1.58A | 1.59B | | 1.60B | | 1.58A | 1.60B | | 1.61B |
|  |  |  | |  | |  |  | |  |
| 3^rd^ Palp Length | 53.05C | 51.59BC | | 59.16D | | 50.91B | 48.98AB | | 48.40A |
| 3^rd^ Palp Width | 20.85AB | 19.80A | | 24.57D | | 20.49A | 23.23CD | | 22.00BC |
| Palpal Ratio | 2.58CD | 2.66D | | 2.46BCD | | 2.45BC | 2.19A | | 2.31AB |
| Length of Flagella 10 | 37.34B | 36.22AB | | 40.89C | | 36.63B | 35.82AB | | 35.18A |
| Length of Flagella 11 | 50.37C | 49.21BC | | 56.52D | | 47.59AB | 47.73ABC | | 45.86A |
| Flagella Ratio* | 1.12B | 1.10AB | | 1.10AB | | 1.09A | 1.11AB | | 1.09AB |
| 5 Apical Segment Length | 302.45B | 296.22AB | | 333.53C | | 289.71A | 290.41AB | | 283.91A |
| 8 Basal Segment Length | 271.09A | 268.76A | | 303.05B | | 265.82A | 263.15A | | 260.30A |
| Segment Ratio* | 1.35ABC | 1.36BC | | 1.39C | | 1.30A | 1.33ABC | | 1.30AB |
|  |  |  | |  | |  |  | |  |
| Larger Spermatheca Length | 48.05BC | 47.39ABC | | 49.29D | | 48.04C | 47.02AB | | 46.46A |
| Larger Spermatheca Width | 33.60CD | 32.83B | | 34.25D | | 33.51C | 32.27AB | | 31.85A |
| Larger Spermatheca Ratio* | 1.43A | 1.45A | | 1.44A | | 1.44A | 1.46A | | 1.46A |
| Smaller Spermatheca Length | 47.541B | 47.21B | | 47.88B | | 47.60B | 46.34A | | 45.89A |
| Smaller Spermatheca Width | 33.22C | 32.34B | | 33.77C | | 33.16C | 31.79AB | | 31.25A |
| Smaller Spermatheca Ratio | 1.43ABC | 1.46BC | | 1.42A | | 1.44AB | 1.46ABC | | 1.47C |
|  |  |  | |  | |  |  | |  |
| Chitinous Plate Length | 12.33BC | 11.55B | | 12.47C | | 12.27C | 10.94AB | | 10.52A |
| Chitinous Plate Width | 18.48B | 17.78B | | 19.67C | | 18.55B | 17.73AB | | 16.56A |
| Chitinous Plate Ratio* | 0.67A | 0.65A | | 0.64A | | 0.67A | 0.62A | | 0.64A |

* indicates non-significant outcome (Bonferroni *P > 0.002*)

**Additional Table 6.** A summary of 15 morphological measurements and eight ratios of *C. scoticus* that exhibited significant differences between sites in the UK, France and Spain.

| **Measurement** | **UK** | | **France** | | **Spain** | | |
| --- | --- | --- | --- | --- | --- | --- | --- |
|  | **Bala** | **Devon** | **Landes** | **Calvados** | **Avià** | **Caldes de Malavella** | |
| Wing Length | 1.39C | 1.36C | 1.37BC | 1.41C | 1.28B | 1.11A |  |
| Wing Width | 0.60C | 0.58C | 0.59C | 0.60C | 0.55B | 0.50A |  |
| Costa Length | 0.87C | 0.84C | 0.86C | 0.88C | 0.79B | 0.67A |  |
| Wing Ratio* | 2.33A | 2.33A | 2.30A | 2.33A | 2.33A | 2.31A |  |
| Costa Ratio | 1.61A | 1.61AB | 1.60A | 1.60A | 1.63BC | 1.65C |  |
|  |  |  |  |  |  |  |  |
| 3^rd^ Palp Length | 53.19C | 51.57BC | 56.42C | 52.20BC | 48.81AB | 47.23A |  |
| 3^rd^ Palp Width | 18.53B | 15.65A | 19.10B | 17.48B | 18.75B | 18.03B |  |
| Palpal Ratio | 2.97AB | 3.40B | 2.98AB | 3.01B | 2.66A | 2.66A |  |
| Length of Flagella 10 | 36.59B | 34.52A | 37.83B | 36.36B | 35.18AB | 34.11A |  |
| Length of Flagella 11 | 50.95A | 49.96A | 54.47B | 50.16A | 49.59A | 48.43A |  |
| Flagella Ratio | 1.15A | 1.15A | 1.16AB | 1.15A | 1.20B | 1.18AB |  |
| 5 Apical Segment Length | 302.32A | 294.30A | 326.89B | 299.86A | 296.44A | 290.68A |  |
| 8 Basal Segment Length | 264.21B | 256.49AB | 282.21C | 260.13B | 247.05A | 247.47A |  |
| Segment Ratio* | 1.40AB | 1.45B | 1.44AB | 1.39A | 1.41AB | 1.42AB |  |
|  |  |  |  |  |  |  |  |
| Larger Spermatheca Length | 61.82BCD | 61.01ABC | 62.35CD | 62.46D | 60.75AB | 60.41A |  |
| Larger Spermatheca Width | 41.56BC | 40.90B | 41.14BC | 42.17C | 40.31A | 39.13A |  |
| Larger Spermatheca Ratio* | 1.49A | 1.50AB | 1.52AB | 1.49A | 1.51AB | 1.55B |  |
| Smaller Spermatheca Length | 61.21BC | 60.46AB | 61.07ABC | 61.81C | 60.32AB | 59.92A |  |
| Smaller Spermatheca Width | 40.78B | 39.93AB | 41.60B | 41.07B | 39.34A | 38.71A |  |
| Smaller Spermatheca Ratio* | 1.51A | 1.52A | 1.47A | 1.51A | 1.54A | 1.56A |  |
|  |  |  |  |  |  |  |  |
| Chitinous Plate Length* | 19.62AB | 19.55AB | 22.30AB | 21.37B | 19.62AB | 18.16A |  |
| Chitinous Plate Width | 21.45BC | 20.13AB | 23.00C | 21.15B | 19.81A | 18.63A |  |
| Chitinous Plate Ratio* | 0.93A | 0.99A | 0.98A | 1.02A | 1.01A | 1.00A |  |

* indicates non-significant outcome (Bonferroni *P > 0.002*)
